# The oxytocinergic system modulates sadistic context-dependent empathy in humans

**DOI:** 10.1038/s41598-017-12671-2

**Published:** 2017-09-29

**Authors:** Siyang Luo, Yiyi Zhu, Ying Xu, Qianting Kong

**Affiliations:** Department of Psychology, Guangdong Key Laboratory of Social Cognitive Neuroscience and Mental Health, Guangdong Provincial Key Laboratory of Brain Function and Disease, Sun Yat-Sen University, Guangzhou, 510006 China

## Abstract

The oxytocinergic system is crucial for sociality and well-being and is associated with empathy. It is suggested that the oxytocinergic system exerts context- and person-dependent effects. We examined how sexual sadistic contexts influenced the effects of the oxytocinergic system on empathic-related behaviors and brain activity in healthy adults. Combining genetic neuroimaging, pharmacological techniques and a psychological paradigm of empathy, we recorded EEG neural responses in female OXTR rs53756 G/G and A/A carriers and measured subjective empathic ratings after intranasal administration of oxytocin/placebo in healthy male adults during the perception of painful facial expressions in sadistic/general social contexts. The results revealed that sadistic contexts modulate oxytocinergic effects on empathy at both behavioral and neural levels. The oxytocinergic system preferentially modulated empathic responses to sadistic contexts. These effects are moderated by individual’s trait empathy. Our combined genetic-pharmacological-imaging results provide a neurochemical mechanism for sadistic context-dependent effects of the oxytocinergic system on empathy.

## Introduction

Empathy, which is the understanding and sharing of the emotional states or conditions of others, plays a vital role in social interaction and benefits human society by promoting prosocial behaviors^1–4^. Impaired empathy may lead to serious social-cognitive dysfunctions, such as psychopathy^5^. Researchers have recently begun to pay more attention to the specific neurochemical systems involved in empathy, such as the oxytocinergic system^6,7^. Both the oxytocin receptor gene (OXTR) and intranasal oxytocin administration (OT) are associated with empathic responses^8,9^, and the experience of empathy, which leads to subsequent generosity toward others, is associated with a 47% increase in oxytocin release^10^.

However, the reported results concerning the effects of the oxytocinergic system on empathy are inconsistent^11^. Several studies that evaluated the effects of oxytocin or OXTR on affect sharing/empathy reported nonsignificant main effects; instead, the effects of the oxytocinergic system on emotional processes were influenced by personal factors or task/stimulus variables^3,12–15^. For example, in the study by Domes *et al*.^16^, oxytocin improved performance on the Reading the Mind in the Eyes Test (RMET) only on more difficult test items. However, oxytocin had no main effect on the accuracy of interpersonal judgments during emotion recognition in other studies; instead, the effect of oxytocin was moderated by individual differences in social proficiency^12^. However, direct experimental evidence that sadistic contexts modulate the effect of the oxytocinergic system on empathy is not yet available. Unlike in non-social contexts (e.g., someone accidentally bumped his head on a door), in the common sadistic context, victims’ suffering was intentionally inflicted by someone else (e.g., one’s body was being beaten with a whip). These sadistic contexts have clear social meanings, suggesting a relationship between the sadist and victims, as well as the subsequent sustained sadistic behavior. The social context in which pain occurs modulates the brain responses to another’s pain^17^. In the sadistic context, sexual sadists lack the ability to empathize with victims^18^, which may lead to an increased likelihood of perpetrating instrumental violence^19^. Thus, an understanding of the functional role of the oxytocin system in modulating the sadistic context-dependent empathic responses may raise important issues regarding the molecular underpinnings of sadistic behavior.

Recently, Ma *et al*.^20^ proposed a social adaptation model (SAM) that provides an integrative understanding of discrepant effects of oxytocin and the modulation of oxytocin action by the personal milieu and context. According to this model, the fundamental function of the oxytocinergic system is to promote social adaptation by modulating emotional responses and adjusting behaviors during social interactions. Humans are social creatures who must maintain a high level of social sensitivity to better adapt to the social environment. Thus, based on accumulating evidence, the social influences of oxytocin vary across social contexts^21,22^, rather than the suggest that oxytocin generally promotes prosociality^23,24^. Therefore, we propose the hypothesis that oxytocin preferentially enhances individuals’ empathy toward others’ suffering (e.g., painful expressions) in sadistic (social salience) contexts in which the individuals simultaneously face various social contexts.

In this study, we primarily focused on the functional effects of the oxytocinergic system on individuals’ empathic responses to others’ suffering in sexual sadistic contexts (vs. general contexts). Compared with painful stimuli in daily contexts, painful stimuli in sexual sadistic contexts show higher social salience and close relationships with participants’ prosocial/antisocial behaviors. As shown in our previous research, both early (92–112 ms) and late (700–1000 ms) empathic neural responses in sexual sadistic contexts, but not in general context, were reduced in female BDSM (B/D (bondage and discipline), D/S (dominance and submission), and S/M (sadism and masochism)) practitioners^18^. In the current study, we further explore the role that oxytocinergic system plays in these sadistic context-dependent empathic responses. We designed two experiments to examine whether the effects of the oxytocinergic system on empathy are influenced by sadistic contexts. Experiment 1 measured empathic neural responses to others’ suffering in different social contexts (sadistic painful, general painful, and general neutral) by recording electroencephalography (EEG) responses in individuals with the homozygous A/A and G/G genotypes of OXTR rs53576. This approach allowed us to examine whether there is an association between OXTR rs53576 and empathic responses and whether this association is modulated by sadistic contexts. Empathic neural responses to others’ suffering were quantified by contrasting perceived painful vs. non-painful expressions, similar to the approach used in previous research^15,25,26^. Experiment 2 further investigated whether intranasal oxytocin interacts with social contexts to affect subjective empathic ratings and whether this sadistic context-dependent oxytocin effect is driven by enhanced empathic responses to painful expressions in sadistic contexts or decreased empathic responses to painful expressions in general contexts.

## Results

### Experiment 1

#### Behavioral results

We conducted ANOVAs of the subjective ratings of self-esteem, life satisfaction and trait empathy. The results did not reveal significant differences between the two genotype groups (F(1,47) = 0.001~1.70, ps = 0.20~0.97, Table S1).

#### Electrophysiological results

**Figure 1:**
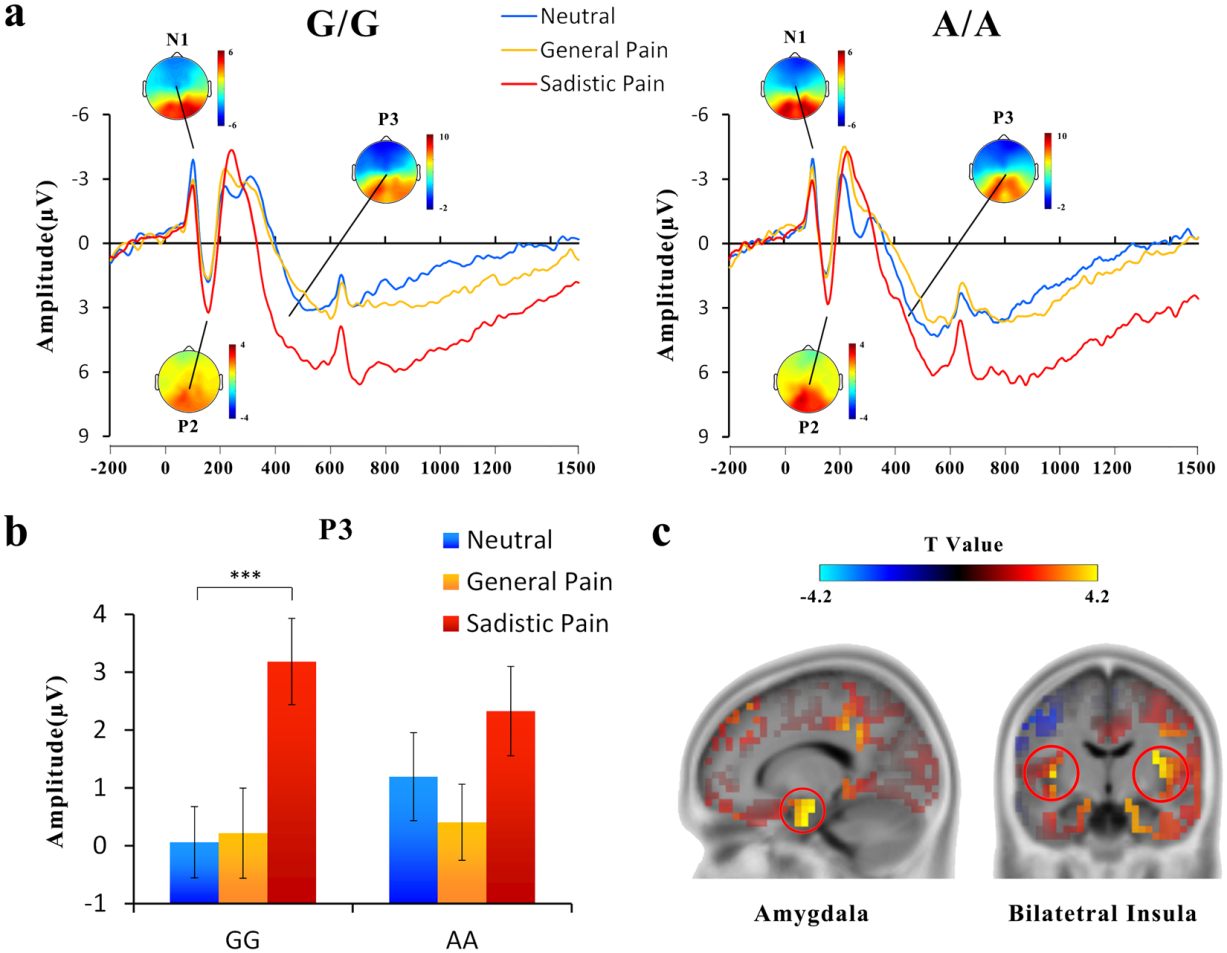
ERP results from Experiment 1. (**a**) ERPs recorded at Fz in response to the general painful, sadistic painful and neutral conditions in the OXTR rs53576 G/G and A/A groups. (**b**) The mean amplitudes at Fz from 300–500 ms (P3). Error bars represent standard errors. (**c**) Illustration of the results of source estimation. Increased activities in response to painful vs. neutral expressions in the P3 time windows were identified in the bilateral amygdala and bilateral insula.

Figure 1a illustrates the ERPs for painful and neutral expressions at a frontal/central electrode. The ERPs were characterized by a negative wave at 88–108 ms (N1) and a positive deflection at 120–180 ms (P2) over the frontal/central areas, which were followed by a negative wave at 200–280 ms (N2) over the frontal/central region and a positive wave (P3) at 300–500 ms over the central area.

The 2 (genotype) × 3 (expression) ANOVAs of the N1 amplitudes showed a significant main effect of expression (F(2,92) = 7.22~7.72, ps ≤ 0.001, η^2^ = 0.13~0.14, Fig. 1a), suggesting that the N1 amplitudes were reduced by painful expressions compared with neutral expressions (neutral vs. sadistic painful, ps = 0.001; neutral vs. general painful, ps < 0.05). Neither the main effect of genotype (F(2,92) = 0.001~0.25, ps = 0.62~0.97, η^2^ = 0.00~0.005) nor its interaction with expression (F(2,92) = 0.45~0.74, ps = 0.48~0.64, η^2^ = 0.01~0.016) was significant for the N1 amplitude, suggesting that the effect of painful expressions on the N1 amplitude was similar for individuals with the G/G and A/A genotype.

The ANOVAs of the P2 amplitudes at 120–180 ms over the frontal/central electrodes showed significant main effects of expression (F(2,92) = 17.35~29.49, ps < 0.001, η^2^ = 0.27~0.39, Fig. 1a), The P2 amplitudes were enlarged by sadistic painful expressions compared with neutral expressions (ps < 0.001). These results replicated the previous ERP findings^25,26^ and suggested that the frontal/central P2 amplitude was involved in coding emotional attributes (i.e., pain). Neither the main effect of genotype (F(2,92) = 0.29~1.19, ps = 0.28~0.60, η^2^ = 0.006~0.03) nor its interaction with expression (F(2,92) = 0.11~0.89, ps = 0.42~0.90, η^2^ = 0.00~0.02) was significant for the P2 amplitude, indicating that the OXTR genotype did not modulate the neural responses of the P2 amplitude to painful expression.

The ANOVAs of the N2 amplitudes at 200–280 ms over the frontal/central electrodes showed significant main effects of expression (F(2,92) = 7.61~12.02, ps ≤ 0.001, η^2^ = 0.14~0.21, Fig. 1a); the N2 amplitudes were enlarged by painful expressions compared with neutral expressions (neutral vs. sadistic painful, ps < 0.005; neutral vs. general painful, ps ≤ 0.005). These results replicate the previous ERP findings^27–29^ and suggest that highly arousing pictures (i.e., pain) elicit larger frontal/central N2 amplitudes than pictures with low levels of arousal. Neither the main effect of genotype (F(2,92) = 0.03~0.33, ps = 0.57~0.86, η^2^ = 0.001~0.007) nor its interaction with expression (F(2,92) = 1.13~1.87, ps = 0.16~0.33, η^2^ = 0.02~0.04) was significant for the N2 amplitude. Thus, the effect of painful expressions on the N2 amplitude was similar for individuals with the G/G and A/A genotypes.

The ANOVAs of the P3 amplitudes showed significant main effects of expression (F(2,92) = 34.68~84.85, ps < 0.001, η^2^ = 0.43~0.65, Fig. 1a), suggesting that the P3 amplitudes were enlarged by painful expressions compared with neutral expressions. Interestingly, the effect was quantified by significant group × expression interactions over the frontal electrodes (FZ, F1-F2, F3-F4, FCZ, FC1-FC2, FC3-FC4: F(2,92) = 2.73~5.30, ps = 0.007~0.07, η^2^ = 0.06~0.10, Fig. 1b). According to a simple effect analysis, the main effects of expression were significant in G/G carriers (F(2,46) = 30.20~57.55, ps < 0.001, η^2^ = 0.57~0.71) but were weaker in A/A carriers (F(2,46) = 8.94~17.62, ps≤0.001, η^2^ = 0.28~0.43). Post hoc analysis further confirmed that the sadistic painful condition (ps < 0.001), but not the general painful condition (ps = 0.85~1.00), was associated with increased P3 amplitudes in the G/G group compared to the neutral condition. For the A/A group, neither the general painful condition nor the sadistic painful condition differed significantly from the neutral condition (general painful vs. neutral: ps = 0.14~0.63; sadistic painful vs. neutral: ps = 0.004~0.17). The simple effect analysis also confirmed that the differences in the amplitudes between sadistic painful and neutral conditions were larger in G/G carriers than in A/A carriers (F(1,46) = 4.00~8.50, ps = 0.005~0.05, η^2^ = 0.08~0.16). Based on the source estimation using sLORETA, the potential sources of the interaction between OXTR and social contexts on empathic responses were the bilateral amygdala and bilateral insula (peak Montreal Neurological Institute (MNI) coordinates: right amygdala: x/y/z = 15/−5/−30, t = 4.214; left amygdala: x/y/z = −15/0/−30, t = 3.795; right insula: x/y/z = 35/−10/15, t = 3.729; left insula: x/y/z = −40/−15/−5, t = 3.788; Fig. 1c).

### Experiment 2

We conducted similar ANOVAs to those described in Experiment 1 on the subjective ratings of self-esteem, SES, life satisfaction and trait empathy, but did not observed significant differences between the two treatment groups (F(1,47) = 0.001~1.70, ps = 0.20~0.97, η^2^ = 0.001~0.05, Table S2).

### Context-dependent oxytocin effects on subjective empathic ratings

**Figure 2:**
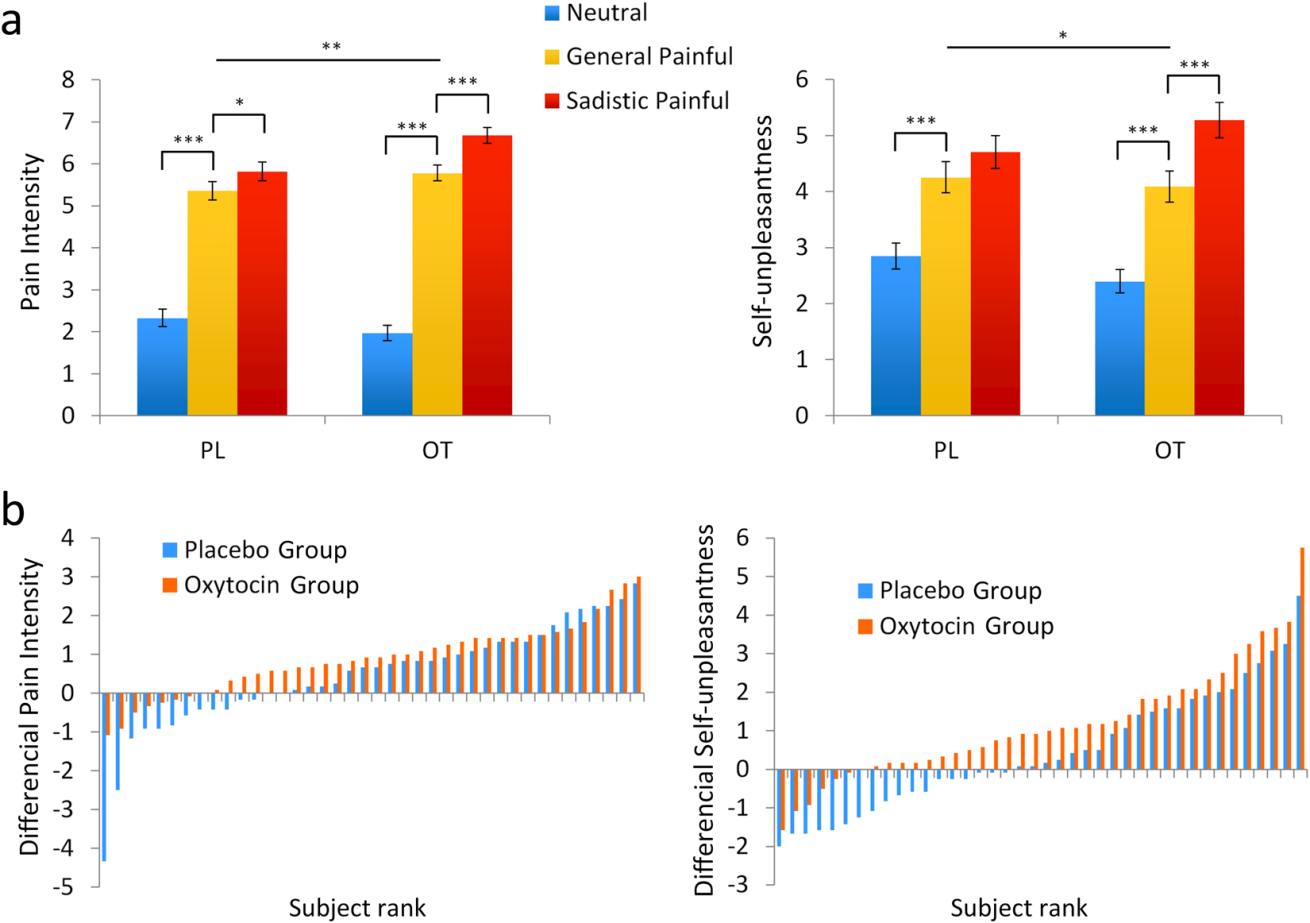
(**a**) Mean pain intensity/self-unpleasantness rating scores in two treatment groups. Error bars represent standard errors. (**b**) Differential pain intensity/self-unpleasantness ratings between the sadistic painful and general painful conditions for each participant.

We conducted ANOVAs on the subjective ratings of pain intensity and self-unpleasantness, with treatment (OT vs. PL) as a between-subjects variable, and expression (neutral, general painful, and sadistic painful) as a within-subjects variable. The result revealed a significant main effect of expression (pain intensity: F(2,156) = 338.76, p < 0.001, η^2^ = 0.81; self-unpleasantness: (F(2,156) = 82.02, p < 0.001, η^2^ = 0.51) and a significant treatment × expression interaction (pain intensity: F(2,156) = 6.70, p = 0.002, η^2^ = 0.08; self-unpleasantness: F(2,156) = 3.94, p = 0.02, η^2^ = 0.05; Fig. 2a). A separate analysis revealed that the main effects of expression were significant in both the OT and PL groups (pain intensity: OT group: F(2,78) = 295.76, p < 0.001, η^2^ = 0.88; PL group: F(2,78) = 100.22, p < 0.001, η^2^ = 0.93; self-unpleasantness: OT group: F(2,78) = 62.17, p < 0.001, η^2^ = 0.61; PL group: F(2,78) = 25.45, p < 0.001, η^2^ = 0.40). The post hoc analysis further confirmed that the pain intensity ratings and self-unpleasantness ratings were higher under the general painful and sadistic painful conditions than under the neutral conditions in both treatment groups (ps < 0.001). In addition, the difference between the general painful and sadistic painful conditions was larger in the OT group (p < 0.001) than in the PL group (pain intensity: p = 0.05; self-unpleasantness: p = 0.19). The differences in both pain intensity ratings between sadistic painful and neutral conditions and between general painful and neutral conditions were enhanced by the OT (sadistic painful vs. neutral: F(1,78) = 10.19, p = 0.002, η^2^ = 0.12; general painful vs. neutral: F(1,78) = 4.80, p = 0.03, η^2^ = 0.06). The differences in the self-unpleasantness ratings between sadistic painful and neutral conditions were also enhanced by the OT (F(1,78) = 5.09, p = 0.03, η^2^ = 0.06), but not the general painful vs. neutral conditions (F(1,78) = 0.81, p = 0.37, η^2^ = 0.01).

We then conducted the non-parametric bootstrap analysis to further determine the significance of the effect of the treatment on context-dependent empathic responses among participants. First, context-dependent empathic responses (the differences in the subjective ratings of pain intensity/self-unpleasantness for general painful vs. sadistic painful conditions) were calculated for each participant. Then, we ranked the OT and PL groups from largest to smallest context-dependent empathic responses and compared the ratings of the two participants at the same rank (e.g., the participant with the largest response in the OT group vs. the participant with the largest response in the PL group). T_Obs_ was calculated from the number of responses that fit the description OT group > PL group, and the results showed that T_Obs_ = 35 for the pain intensity ratings and T_Obs_ = 40 for the self-unpleasantness ratings. These scores differ from the null hypothesis distribution (pain intensity: p = 0.07; self-unpleasantness: p = 0.009, Fig. 2b), suggesting that the differences in pain intensity and self-unpleasantness ratings observed between the general painful and sadistic painful conditions among participants were enhanced by the OT.

### Effects of oxytocin on sadistic painful vs. general painful conditions

We directly compared pain intensity scores between the OT and PL groups to clarify whether the observed effect of the treatment on context-dependent empathic responses was driven by enhanced empathic responses to sadistic painful expressions or decreased empathic responses to general painful expressions. The pain intensity rating scores for sadistic painful conditions were higher in the OT group than in the PL group (p < 0.005), but these group differences were not observed for the neutral and general painful conditions (ps > 0.1). A similar pattern was also observed for the self-unpleasantness rating scores; however, the observed difference was not significant (ps > 0.05).

**Figure 3:**
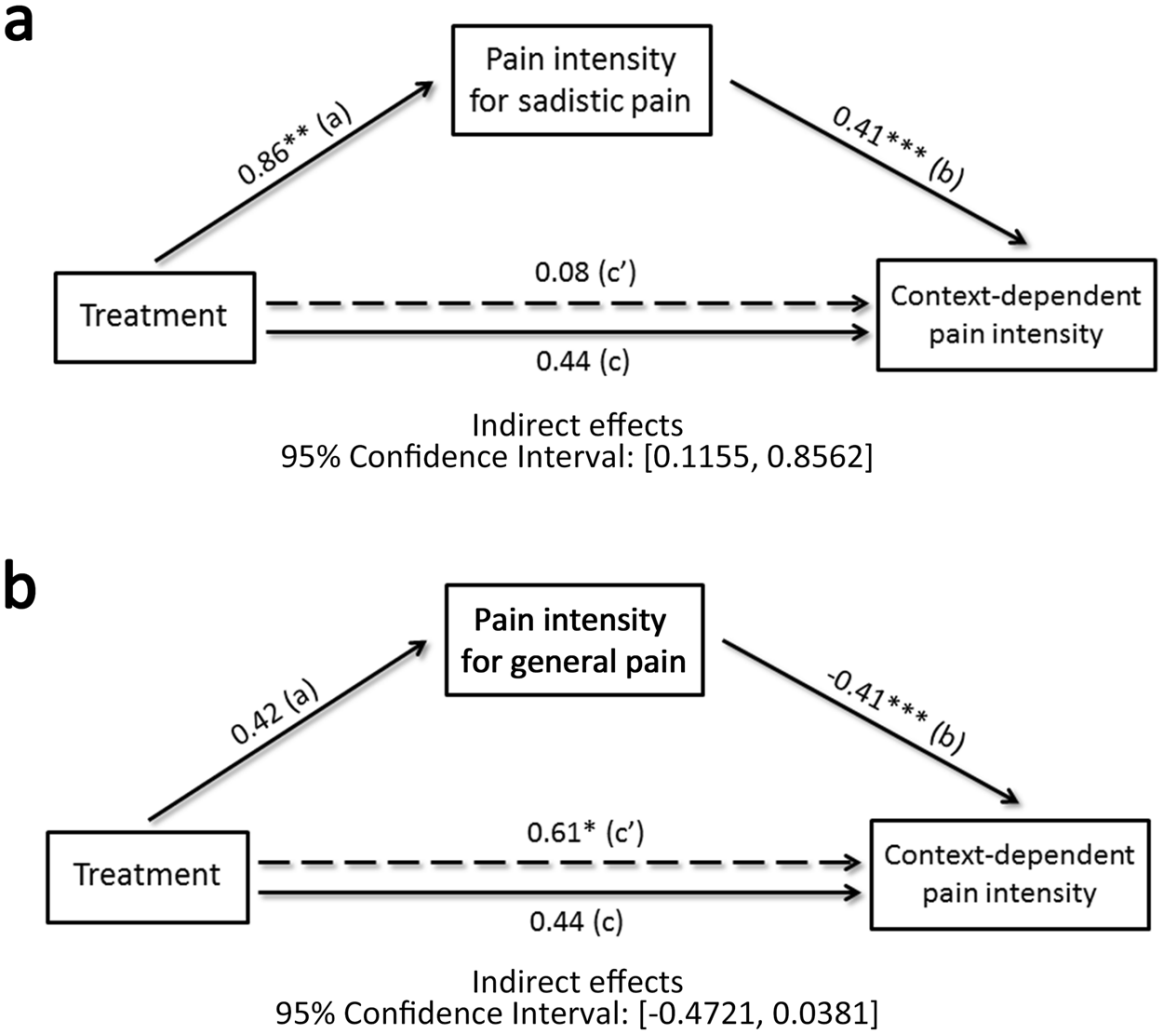
Illustration of the mediation effect. (**a**) The effect of treatment on context-dependent pain intensity ratings was significantly reduced when the pain intensity ratings for sadistic painful was included in the regression model. (**b**) The effect of treatment on context-dependent pain intensity ratings did not differ significantly when the pain intensity ratings for general painful was included in the regression model.

We then conducted mediation analysis to further test whether the effect of the treatment on context-dependent empathic responses was mediated by its effect on sadistic painful conditions, but not general painful conditions. The bootstrap resampling analysis revealed a significant effect of treatment (IV) on the pain intensity rating of sadistic painful conditions (mediator) (B = 0.86, t(80) = 2.99, p < 0.005) and a significant effect of the mediator on context-dependent pain intensity ratings (DV) (B = 0.41, t(80) = 4.49, p < 0.001). The indirect effect of the IV on the DV through the mediator differed from zero with 95% confidence (95% confidence interval (CI): [0.1155, 0.8562], Fig. 3a and Supplementary Table S3). Similar analyses were conducted with the pain intensity ratings of general painful conditions as the mediator, and the effect of the mediator on context-dependent pain intensity ratings was significant (DV) (B = −0.41, t(80) = −4.48, p < 0.001), whereas the effect of the treatment (IV) on the mediator was not significant (B = 0.42, t(80) = 1.47, p = 0.15). Moreover, the indirect effects failed to differ from zero with 95% confidence (95% CI: [−0.4721, 0.0381], Fig. 3b and Supplementary Table S4). Thus, the observed effect of oxytocin on context-dependent empathic responses was primarily derived from influences on the participants’ ratings for sadistic painful expressions.

### Effects of oxytocin on empathic responses and alert responses

Next, we tested whether the effect of the treatment on context-dependent empathic responses resulted from differences in alert responses (arousal and perceived threat) between the general painful and sadistic painful expressions. ANOVAs of the subjective arousal ratings revealed significant main effects of expression (F(2,156) = 45.85, p < 0.001, η^2^ = 0.37) and treatment (F(1,78) = 5.43, p = 0.02, η^2^ = 0.07). The OT group showed weaker arousal responses than the PL group. The treatment × expression interaction was also significant (F(2,156) = 3.38, p = 0.04, η^2^ = 0.04). A separate analysis revealed significant main effects of expression in both the OT and PL groups (OT group: F(2,78) = 15.08, p < 0.001, η^2^ = 0.28; PL group: F(2,78) = 31.94, p < 0.001, η^2^ = 0.45). According to the post hoc analysis, the arousal ratings for sadistic painful conditions were lower in the OT group than in the PL group (p < 0.005). These group differences were not observed for the neutral and general painful conditions (ps > 0.1). These patterns differed from those observed for the pain intensity rating that the OT group recorded higher ratings for sadistic painful conditions than the PL group.

ANOVAs of the subjective perceived threat ratings revealed a significant main effect of expression (F(2,156) = 87.00, p < 0.001, η^2^ = 0.53). However, neither the main effect of treatment (F(1,78) = 0.001, p = 0.97, η^2^ = 0.00) nor its interaction with expression (F(2,156) = 2.54, p = 0.08, η^2^ = 0.03) was significant. The post hoc analysis further confirmed that the perceived threat ratings for the general painful and sadistic painful conditions were higher than the threat ratings for the neutral condition (p < 0.001), and the perceived threat ratings for the sadistic painful condition were higher than for the general painful condition in both treatment groups (p < 0.001). Thus, the differential arousal and perceived threat ratings between the sadistic painful and general painful conditions were not enhanced by the OT, and the effect of OT on context-dependent empathic responses was not simply induced by enhanced arousal and the perceived threat of the sadistic painful expressions.

### Modulatory role of trait empathy on the effect of oxytocin

**Figure 4:**
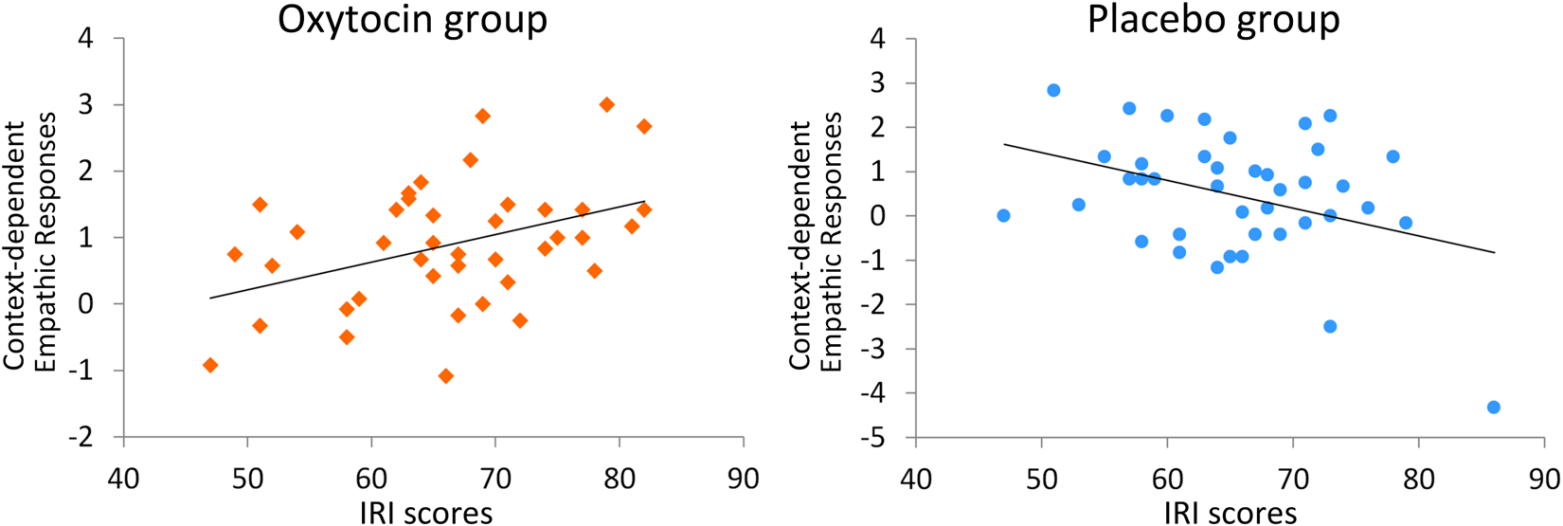
Association between trait empathy and context-dependent empathic responses in the two treatment groups. The participants’ context-dependent empathic responses correlated positively with IRI scores in the OT group. The PL group showed the opposite pattern.

Finally, we tested whether the effects the treatment on context-dependent empathic responses were moderated by the participants’ general empathic abilities. According to the regression analysis, the context-dependent empathic responses of the participants in the OT group correlated positively with IRI scores (r(40) = 0.41, p < 0.01, Fig. 4). The PL group did not show a similar pattern (r(40) = −0.37, p = 0.02, Fig. 4; after removing one multiple outlier: r(39) = −0.18, p = 0.27). Hierarchical regression analyses further confirmed that the interaction of treatment and IRI scores reliably predicted participants’ context-dependent empathic responses (β = 0.38, t = 3.60, p = 0.001; Supplementary Table S5), suggesting that the effects of oxytocin on context-dependent empathic responses were modulated by individuals’ empathic abilities. Moreover, an effect of oxytocin on the differing empathic responses to different social contexts was primarily observed in individuals with strong empathic abilities.

Further analysis also confirm that the context-dependent effects of oxytocin on pain intensity and self-unpleasantness ratings were not driven by differences in the intensity of sadistic painful and general painful stimuli (See Supplementary results and Table S6 and Figures S1 and S2).

## Discussion

Current research has focused on elucidating the sadistic context-dependent effects of the oxytocinergic system on empathy in healthy volunteers. We have provided genetic, imaging and pharmacologic evidence that both OXTR and intranasal oxytocin interact with the sadistic context to modulate neural activity and subjective responses related to empathic processes. Specifically, in Experiment 1, the P3 amplitude was enlarged in response to sadistic painful stimuli (vs. neutral) but not in response to general painful stimuli in individuals with the G/G genotype of OXTR rs53576, and this difference was not observed in A/A carriers. Furthermore, in Experiment 2, intranasal oxytocin (vs. placebo) increased context-dependent empathic responses driven by enhanced empathic responses to sadistic painful stimuli, but not by decreased empathic responses to general painful stimuli. Thus, the genetic structure of the oxytocinergic system and relevant molecules play important roles in empathic processes in healthy adults, and this effect may be moderated by different contexts. These results provide neuro-pharmaco-genetic evidence that improves our understanding of the previous inconsistent results describing how the oxytocinergic system modulates subjective feelings and neural responses related to empathy.

Based on our EEG results, the sadistic context modulated the relationship between the OXTR and empathic neural responses, as indicated by P3 amplitudes. The P3 amplitude is a controlled component that is used as a neurophysiological indicator of emotional evaluation^30–33^. This indicator appears to be sensitive to both the intrinsic affective properties of the stimulus and the local affective context in which the stimulus is presented^34,35^. As shown in the study by Sessa *et al*.^36^, painful contexts, but not painful expressions, selectively modulated the P3 amplitude when contexts and expressions were presented. Consistent with these findings, our results suggested that the OXTR genotype may influence context-dependent empathic neural responses, and G allele carriers showed higher sensitivity to social contexts that included painful stimuli than A/A homozygotes.

In early intranasal oxytocin studies, researchers regarded oxytocin as the “prosocial hormone”, based on evidence showing that intranasal oxytocin promotes prosocial behaviors such as trust. However, in other studies using specific contexts, intranasal oxytocin also increased antisocial behaviors such as envy^22^. Accumulating evidence suggests that the effects of the oxytocinergic system on emotional processes are influenced by personal factors or task contexts^3,12–15^. For example, in the study by Hurlemann *et al*.^13^ using the feedback-guided item-category association task, participants’ exhibited higher learning performance when social rather than non-social reinforcement was used. Moreover, an intranasal OT potentiated this social reinforcement advantage by enhancing social reinforcement learning but not decreasing non-social reinforcement learning, suggesting a neural basis of the effects of oxytocin on the facilitation of social salience. Consistent with these findings, oxytocin increased the differential subjective pain intensity and self-unpleasantness ratings between various social contexts in our study. Furthermore, the mediation analysis confirmed that the effect of oxytocin on context-dependent empathic responses was mainly due to enhanced ratings for a social salience context (sadistic painful) but not decreased ratings for a general context (general painful). Other researchers have proposed that attentional orientation to social stimuli, like the eye regions, was enhanced by the increased salience of social cues after intranasal oxytocin administration^37^. Thus, OT plays a general role in increasing the salience of social cues and is sensitive to social contexts and individual differences. Moreover, the OT has important roles in processing social information and in allowing individuals to prepare for social engagement and social consequences^20^.

Furthermore, we did not observe a similar context-dependent effect of oxytocin on arousal and perceived threat. OT treatments significantly decreased arousal ratings for sadistic painful stimuli, but not perceived threat ratings, compared to the PL treatments, suggesting that the sadistic context-dependent modulation of the effect of oxytocin on subjective empathic responses was not simply attributed to general alert responses. Similarly, the intranasal administration of oxytocin specifically improves recognition memory for faces but not for non-social stimuli, and the effects did not covary with arousal^38^. This finding is consistent with the SAM of the oxytocinergic system, which suggests that the fundamental function of the oxytocinergic system is to promote social adaptation by modulating emotional responses and adjusting behaviors during social interactions, rather than simply enhancing alert responses.

Our genetic imaging and pharmacologic findings have important implications. First, recent studies have suggested a vital effect of the oxytocinergic system on empathy, but have reported inconsistent results, such as a lack of significant main effects of oxytocin or OXTR on affect sharing/empathy^3,12–15^. In the current study, we did not observe significant main effects of the OXTR genotype and intranasal oxytocin on empathic neural responses and behavioral tendencies. However, the effects were modulated by social contexts, suggesting that social salience information plays an important role in regulating the effects of the oxytocinergic system on social affective processes. Our findings imply that the oxytocinergic system evaluates the social salience levels of various contexts and preferentially modulates affective processes in high-social-salience contexts.

Second, sexual sadists have been reported to lack the ability to empathize with victims, which may lead to an increased likelihood of perpetrating instrumental violence^19^. However, in other studies, sexual sadists did not differ from non-sadistic sexual offenders with regard to emotional empathy for either positive or negative stimuli^39^. The sadistic context in our studies, in which the suffering of victims was intentionally inflicted by someone else, has clear social meanings and suggests a relationship between the sadist and victims, as well as the subsequent sustained sadistic behavior. As shown in our previous studies, both early (92–112 ms) and late (700–1000 ms) empathic neural responses in sexual sadistic contexts, but not in general context, were reduced in female BDSM practitioners^18^, suggesting that BDSM practitioners may have weaker ability to empathize with the suffering of others in sexual sadistic contexts, but not in general contexts. The results of the current study further suggest a functional role for the oxytocin system in these context-dependent empathic responses, as intranasal oxytocin enhanced these context-dependent empathic responses. Taken together, these results provide a possible explanation for the previous discrepancies in the results describing the empathic abilities of sexual sadists. The oxytocinergic system of sexual sadists may function abnormally, preventing them from effectively empathizing with the suffering of others in social salience contexts (such as sexual assault), in which sexual sadists are more likely to commit crimes than in common contexts. However, exogenous oxytocin (e.g., intranasal oxytocin) may potentially function to buffer these context-dependent weaken empathic responses in sexual sadists. This theoretical framework should be examined in the future by exploring the relationship between the function of the oxytocinergic system and various social contexts in psychopathic participants.

A potential limitation of the current study is the lack of female participants in the intranasal oxytocin study, which prevents us from testing whether social context modulates the effect of oxytocin on empathic responses similarly in males and females. An intranasal OT was recently shown to produce significant gender-specific effects^40,41^. Although the social context interacts with the OXTR genotype to modulate empathic neural responses in female participants, we did not determine whether a significant intranasal oxytocin × gender interaction exists for social context-dependent empathic responses.

In this study, we did not examine the effects of intranasal oxytocin on different OXTR genotypes. To date, no direct evidence indicates whether the distribution or activity of the receptor in the central nervous system differs between individuals with the A/A and G/G OXTR genotypes. Combined with intranasal oxytocin administration, future experiments investigating this question could more deeply explore the molecular mechanism by which the OXTR genotype influences social emotional processes.

## Conclusions

While interest in the identification of neurochemical mechanisms underlying human social emotional processes has increased, our findings illuminate how social environmental factors interact with oxytocinergic biological factors to modulate emotional processes. Our results suggest that the impact of the oxytocinergic system on empathy depends on the social context and that a high-social-salience context (sexual sadistic) may induce the oxytocinergic system to preferentially enhance the emotional processes of individuals.

## Methods

### Participants

Informed consent was obtained from all participants prior to participation, and all participants were debriefed after the experiments. This study was approved by the local ethics committee of the Department of Psychology of Sun Yat-sen University. All experiments were performed in accordance with relevant guidelines and regulations.

#### Experiment 1

Although previous research found that the endogenous oxytocin level changed during experimental manipulation in both females and males^42^, some research further compared the gender difference and suggested that the endogenous oxytocin level changed larger during social relevant tasks in females than in males^43,44^. For example, serum oxytocin levels increased statistically more for women who interacted with their dog when compared with women in the reading control condition. There was no significant increase in oxytocin level in men after interaction with the bonded dog compared with the reading control condition^43^. Because OXTR encodes the oxytocin receptor, individuals with different OXTR genotypes may show larger differences among subjects whose oxytocin level shows higher sensitivity to social context. Therefore, all participants in this study were females. Forty-eight Chinese female adults aged 19–30 years (mean (M) = 21.66 years, standard deviation (SD) = 2.45) participated in this study as paid volunteers. The ages of the 24 G/G individuals (M = 21.40 years, SD = 2.24) and the 24 A/A individuals (M = 21.92 years, SD = 2.68) were comparable. All participants were right-handed, had normal or corrected-to-normal vision, did not have a history of abnormal neurological symptoms, and were not menstruating, pregnant, or using hormonal drugs. No significant differences in self-esteem, life satisfaction or trait empathy were observed between the two genotype groups (see Table S1 for details). The OXTR rs53576 polymorphism was genotyped using the TaqMan genotyping platform (see the online supplementary material for DNA isolation and analysis procedures).

#### Experiment 2

Because males show a steadier endogenous oxytocin level and less changes during social relevant tasks compared with females^43,44^, which may lead to a more significant and robust effect of intranasal oxytocin, most previous studies using intranasal oxytocin chose males as their participants. Consistent with previous intranasal oxytocin studies, all participants in the Experiment 2 were male. Eighty Chinese male adults aged 18–26 years (M = 19.76, SD = 1.38) participated in this study as paid volunteers. The participants were randomly assigned to the oxytocin treatment (OT) or placebo treatment. Each group comprised 40 participants. No age difference existed between the OT and placebo groups (F(1, 79) = 0.06, p = 0.81, η^2^ = 0.001). All participants were right-handed, had normal or corrected-to-normal vision, and did not have a history of abnormal neurological symptoms. No significant differences in self-esteem, life satisfaction or trait empathy were observed between the two genotype groups (see Table S2 for details).

### Materials and Procedure

#### Experiment 1

**Stimuli and procedure:** The stimuli used during the EEG recording were consistent with a previous study^18^, and consisted of 48 digital photographs of female faces with neutral or painful expressions, including 16 neutral expressions in general contexts (general neutral, with no painful or sexual sadistic information involved), 16 painful expressions in general contexts (general painful) and 16 painful expressions in sexual sadistic contexts (sadistic painful, e.g. a female model showing a painful expression when her body was being beaten with a whip) (see the online supplementary material for a detailed description of the selection criteria for the stimuli). Presentation 0.73 safeware was used to program and display the stimuli. During the EEG recording, each face was displayed with a visual angle of 4.7 × 4.7° (width × height: 9.92 × 9.92 cm) at a viewing distance of 120 cm in the center of a gray background for 500 ms. The interstimulus intervals consisted of a fixation cross with a duration that varied randomly between 800 and 1400 ms. The participants completed 4 EEG blocks during the experiment. A total of 96 trials were included in each block. Each photo was presented 2 times in a random order in each block. The participants made judgments about the expression of each face (painful vs. neutral) and pressed a button with their right index or middle fingers.

After the EEG recording, the participants completed the Interpersonal Reactivity Index (IRI)^45^, which measures empathy traits. The original 28-item questionnaire consists of four 7-item subscales (Fantasy, Perspective Taking, Empathic Concern and Personal Distress), which measure separate but intercorrelated components of empathy. The items were presented as statements, and the participants rated their agreements on a 5-point Likert-type scale (1 = does not describe me well, 5 = describes me well). The participants were also asked to complete the Rosenberg Self-Esteem Scale^46^ and the Satisfaction with Life Scale^47^ to estimate their self-esteem and life satisfaction, respectively.

**EEG recording and analysis:** The EEG recordings were collected from 64 scalp electrodes based on the 10/20 system, as well as two electrodes placed on the left and right mastoids. All data were re-referenced off-line to an average mastoid reference. Eye blinks and vertical eye movements were monitored with electrodes located above and below the left eye. The horizontal electrooculogram was recorded from electrodes placed 1.5 cm lateral to the left and right external canthi. The EEG was amplified (bandpass 0.1–100 Hz) and digitized at a sampling rate of 250 Hz. The event-related potentials (ERPs) in each condition were averaged separately off-line, with an epoch beginning 200 ms before stimulus onset and continuing for 1500 ms. Trials that were contaminated by eye movements, muscle potentials exceeding ± 50 µV at any electrode and response errors were excluded from the averages. This approach resulted in the rejection of 22.8 ± 11.9% of the trials. The baseline for the ERP measurements was the mean voltage of a 200 ms pre-stimulus interval, and latency was measured relative to the stimulus onset. The mean amplitudes of each ERP component were calculated at electrodes selected from the frontal (Fz, F1, F2, F3, F4, FCz, FC1, FC2, FC3, and FC4), central (Cz, C1, C2, CPz, CP1, and CP2), parietal (Pz, P1, and P2), and occipito-temporal (PO7, PO8, P7, and P8) regions. The analysis of variance (ANOVA) tests of the mean ERP amplitudes recorded at the bilateral electrodes included the hemisphere (electrode over the left vs right hemisphere) as a within-subjects variable. We used the false discovery rate (FDR) correction for multiple comparisons.

Both voltage topography and standardized Low Resolution Brain Electromagnetic Tomography (sLORETA)^48^ were used to estimate potential sources of empathic neural responses (see the online supplementary material for details).

### Experiment 2

**Oxytocin administration:** The procedure used to administer oxytocin and the placebo was similar to the procedures reported in previous studies^24,49,50^. A single intranasal dose of 24 IU of oxytocin (OT) or placebo (PL) (containing the active ingredients but not the neuropeptide) was self-administered by nasal spray approximately 40 min before the experimental task under experimenter supervision. The spray was administered to participants three times, and each administration consisted of one inhalation of 4 IU into each nostril. In a double-blind, placebo-controlled, between-subjects design, 10 groups of 4 participants were randomly assigned to the placebo treatment, and the other 10 groups of 4 participants were assigned to the OT treatment. A group of 4 participants who were performing the experiment at the same time was assigned to the same treatment (oxytocin or placebo) to avoid potential influences of oxytocin or placebo between individuals.

**Stimuli and procedure:** We estimated participants’ self-esteem, SES, life satisfaction, and trait empathy using the same measurements as described in Experiment 1, which aimed to identify any differences in participants’ basic psychological states and trait empathy between the two treatment groups. Then, the participants were administered OT or PL and asked to perform the empathic rating task 40 min later. The participants were required to rest during this 40-min period, consistent with previous intranasal oxytocin research. The rating stimuli used for this experiment were the same as in Experiment 1. Presentation 0.73 sofeware was used to program and display the stimuli. The participants evaluated each photograph along the following four dimensions on a 9-point Likert scale (1 = not at all, 9 = extremely strong): 1) pain intensity, 2) self-unpleasantness, 3) threat, and 4) arousal. The pain intensity ratings reflect the subjective empathic responses of the participants, self-unpleasantness reflects the subjective feelings of the participants during empathy processes, and the threat and arousal ratings reflect the subjective alert responses of the participants. In addition, the participants also made judgments about whether each photograph was realted to sexual sadism on a 9-point Likert scale (1 = not at all related to sexual sadism, 9 = absolutely related to sexual sadism).

**Mediation analysis:** Similar to our previous studies^51^, a resampling method known as bootstrapping was used to establish mediation^52^. Mediation analyses were conducted to examine whether the treatment effect on context-dependent empathic responses occurs through enhanced empathic responses to sadistic painful expressions or decreased empathic responses to general painful expressions (see the online supplementary material for details).

**Non-parametric bootstrap analysis:** Non-parametric bootstrap analyses were conducted to further determine the significance of the effect of the treatment on context-dependent empathic responses^53^ (see the online supplementary material for details).

**Moderation analysis:** Hierarchical regression analyses were conducted to examine whether empathy traits moderated the effect of the treatment (as the independent variable (IV)) on context-dependent empathic responses (as the dependent variable (DV)) (see the online supplementary material for details).

## Electronic supplementary material

supplementary information
