# Supplementary material for: The oxytocinergic system modulates sadistic context-dependent empathy in humans
Source: Sci Rep. 2017 Sep 29;7:12463. doi: 10.1038/s41598-017-12671-2 (PMC5622090; doi:10.1038/s41598-017-12671-2)
Supplement: Supplementary file 1 — supplementary information [file 41598_2017_12671_MOESM1_ESM.doc]

**Supplementary Information**

**The oxytocinergic system modulates sadistic context-dependent empathy in humans**

**Siyang Luo^1,*^, Yiyi Zhu^1^, Ying Xu^1^, Qianting Kong^1^**

**^1^Department of Psychology,**

**Guangdong Provincial Key Laboratory of Social Cognitive Neuroscience and Mental Health,**

**Guangdong Provincial Key Laboratory of Brain Function and Disease,**

**Sun Yat-Sen University**

**Guangzhou 510006, China**

**Supplementary information:**

**9 supplementary sections**

**Table S1-6**

**Figure S1**

**Experiment 1**

**Methods**

*Stimuli*

Before our experiment, 20 students who majored in psychology rated 72 original pictures along 5 dimensions: 1) pain intensity, 2) unpleasantness, 3) enjoyment, 4) arousal and 5) relation to sexual sadism. The 16 pictures that were used for the general painful condition had the highest pain intensity and lowest sexual sadism-related scores, and the 16 pictures that were used in the sadistic painful condition had the highest pain intensity and highest sexual sadism-related scores. Therefore, in our experiments, the pain intensities of the pictures that were used for both the general painful and sadistic painful conditions were higher than the pain intensities of the pictures that were used for the neutral condition. Similarly, the sexual sadism-related rating scores of the pictures that were used for the sadistic painful condition were higher than the sexual sadism-related rating scores of the pictures that were used for the neutral and general painful conditions.

*Genotyping*

OXTR rs53576, which is located in the third intron of OXTR, was selected for genotyping. Among all single-nucleotide polymorphisms (SNPs) in OXTR that have been reported to be related to social emotional sensitivity, rs53576 has the most evidence. Hence, in the current study, we focus mainly on this SNP. This SNP was genotyped by using the TaqMan genotyping platform. The TaqMan probes were ordered from the Assays on Demand system of the Applied Biosystems (Applied Biosystems, Foster City, CA, USA, [http://www.appliedbiosystems.com](http://www.sciencedirect.com/science?_ob=RedirectURL&_method=externObjLink&_locator=url&_issn=03043940&_origin=article&_zone=art_page&_plusSign=%2B&_targetURL=http%253A%252F%252Fwww.appliedbiosystems.com%252F)). Genotyping was performed in 5-µl system containing 2.5 µl of TaqMan Universal PCR Master Mix, 0.25 µl of 20× TaqMan probe and 1 µl of genomic DNA using Roche LightCycler 480 II (Roche Diagnostics, Beijing, China). Allele calling was performed using LightCycler CW 1.5 software (Roche Diagnostics).

*EEG recording and analysis*

Both voltage topography and sLORETA^1^ were used to estimate potential sources of empathic neural responses. sLORETA is a linear method for computing statistical maps from EEG data that reveal locations of the underlying source processes and do not require a priori hypotheses regarding the field distribution of the active sources. We performed the sLORETA analysis to assess the 3D current source of neural activity that differentiated between ERPs for pain and neutral expressions. A boundary element model was first created with approximately 5000 nodes from a realistic head model. Statistical nonparametric maps were calculated in a specific time window to estimate the source that differentiated ERPs for pain and neutral expressions. The log of the F ratio and t values of averages were used and considered with a 0.95 level of significance.

**Experiment 2**

**Methods**

*Oxytocin administration.*

The procedure used to administer oxytocin and the placebo was similar to previous study showing significant effects of oxytocin on psychological tendencies or social behaviors^2, 3, 4^. A single intranasal dose of 24 IU of OT or PL (containing the active ingredients but not the neuropeptide) was self-administered by nasal spray approximately 40 min before the experimental task under experimenter supervision. The spray was administered to participants three times, and each administration consisted of one inhalation of 4 IU into each nostril. In a double-blind, placebo-controlled, between-subject design, 10 groups of 4 participants were randomly assigned to the placebo treatment, and the other 10 groups of 4 participants were assigned to the oxytocin treatment. A group of 4 participants who were performing the experiment at the same time was assigned to the same treatment (oxytocin or placebo) to avoid potential influences of oxytocin or the placebo between individuals.

*Stimuli and procedure*

The participants first completed the Rosenberg Self-Esteem Scale^5^, Subjective SES test^6^, the Satisfaction with Life Scale^7^ and the Interpersonal Reactivity Index Scale^8^ to estimate their self-esteem, SES, life satisfaction, and trait empathy, respectively. These measurements aimed to compare if potential differences in participants’ basic psychological states and trait empathy between different treatment groups. Then, the participants were administered OT or PL and asked to perform the empathic rating task 40 min later. The rating stimuli for this experiment were the same as those used in Experiment 1. The participants evaluated each photograph along four dimensions: 1) pain intensity, 2) self-unpleasantness, 3) threat and 4) arousal on a 9-point Likert scale (1=not at all, 9=extremely strong). Pain intensity ratings reflect participants’ subjective empathic responses, self-unpleasantness reflects the participants’ subjective feelings during empathy processes, and threat and arousal ratings reflects the participants’ subjective alert responses. In addition, the participants also made judgments about whether each photograph was related to sexual sadism on a 9-point Likert scale (1=not at all related to sexual sadism, 9=absolutely related to sexual sadism).

*Mediation analysis*

Mediation analyses were conducted to examine whether the effect of the treatment on context-dependent empathic responses occurs through enhanced empathic responses to sadistic painful expressions (rather than decreased empathic responses to general painful expressions). Similar to our previous studies^9^, a resampling method known as bootstrapping was used to establish mediation^10^. Bootstrapping is a non-parametric approach to estimate the effect-size and test the hypothesis that is increasingly recommended for many types of analyses, including mediation^11, 12^. Rather than imposing questionable distributional assumptions, bootstrapping generates an empirical approximation of the sampling distribution of a statistic by repeated random resampling of the available data, and uses this distribution to calculate p-values and construct conﬁdence intervals (5000 resamples were taken for these analyses). Moreover, this procedure supplies superior conﬁdence intervals (CIs) that are bias-corrected and accelerated^13^.

*Non-parametric bootstrap analysis*

The non-parametric bootstrap analysis was applied to further determine the significance of the effect of the treatment on context-dependent empathic responses ^14^. First, context-dependent empathic responses (the differences in subjective ratings of pain intensity/self-unpleasantness for sadistic painful vs. general painful conditions) were calculated for each participant. For the null hypothesis (H0) distribution, data from all 80 participants were bootstrapped, and consisted of A and B groups with 40 people in each group. The context-dependent empathic responses of participants in groups A and B were ranked from the largest to the smallest values. Then, we compared the ratings of the two participants with the same ranks in groups A and B (e.g., the largest rank in group A vs. the largest rank in group B), and counted the number of A≥B (the theoretical distribution ranged from 0-40) as the result of one bootstrap (T_Null_). Ten thousand bootstrap resamples were used for the null hypothesis distribution. We then calculated the number of pain intensity and self-unpleasantness ratings of participants in oxytocin group that were greater than of the ratings of participants in the placebo group (T_Obs_) using the same method and located T_Obs_ in the null hypothesis distribution. The achieved significance level (ASL) was calculated as follows:

ASL = Prob_H0_{ T_Null_≥u_Obs_}

ASL_boot_ = #{ T_Null_≥u_Obs_}/B

where T_Null_ reflects the result of each null hypothesis bootstrapping procedure and T_Obs_ reflects the result of the comparison between the OT and PL groups, in which B represents the number of bootstraps^14^.

*Moderation analysis*

Hierarchical regression analyses were conducted to examine whether empathy traits moderated the treatment (IV) effect on context-dependent empathic responses (DV). The IV (treatment) and the moderator (IRI scores) were normalized before the hierarchical regression analysis. The interactions between treatments and IRI scores were calculated by multiplying the normalized variables^15^. Normalized treatment, IRI scores and their interactions were then sequentially entered into the hierarchical regression analysis. The moderator effect was indicated by a significant interaction of treatment and IRI scores on individuals’ context-dependent empathic responses.

**Results**

We calculated the pain intensity scores for each sadistic painful stimulus and general painful stimulus to further confirm that the context-dependent effects of oxytocin on pain intensity and self-unpleasantness ratings were not driven by differences in the intensity of sadistic painful and general painful stimuli. For sadistic painful stimuli, the stimuli of the top four high pain intensity scores were regarded as the high-intensity condition, and the stimuli of the top four low scores were regarded as the low-intensity condition. We then conducted ANOVAs on the subjective ratings of pain intensity and self-unpleasantness under the sadistic painful condition, with the treatment (OT vs. PL) as a between-subject variable and pain intensity (low vs. high) as a within-subject variable. If the effects observed in our study were not context-specific, but rather reflected the more intense stimuli, then we should observe a significant treatment×pain intensity interaction in these analyses. However, the interactions between treatment and pain intensity were not significant for either pain intensity or self-unpleasantness ratings (pain intensity: F(1,78)=0.80, p=0.374, η^2^=0.01; self-unpleasantness: F(1,78)=2.07, p=0.154, η^2^=0.03; & Figure S1). Similar analyses were conducted for the general painful condition, and the interactions between treatment and pain intensity were not significant for either pain intensity or self-unpleasantness ratings (pain intensity: F(1,78)=1.17, p=0.284, η^2^=0.02; self-unpleasantness: F(1,78)=0.19, p=0.664, η^2^=0.002; Figure S1). Thus, the context-dependent effects observed in our study were not due to a simple difference in intensity.

We further conducted ANOVAs on the subjective ratings of pain intensity and self-unpleasantness, with treatment (OT vs. PL) as the between-subjects variable, and expression (neutral, general painful, and low intensity sadistic painful) as the within-subjects variable. The result revealed a significant main effect of expression (pain intensity: F(2,156)=254.62, p<0.001, η^2^=0.77; self-unpleasantness: (F(2,156)=57.96, p<0.001, η^2^=0.43) and a significant treatment × expression interaction (pain intensity: F(2,156)=7.43, p=0.001, η^2^=0.09; self-unpleasantness: F(2,156)=3.40, p=0.036, η^2^=0.04). A separate analysis revealed that the main effects of expression were significant in both the OT and PL groups (pain intensity: OT group: F(2,78)=233.24, p<0.001, η^2^=0.86; PL group: F(2,78)=72.00, p<0.001, η^2^=0.65; self-unpleasantness: OT group: F(2,78)=41.49, p<0.001, η^2^=0.52; PL group: F(2,78)=19.29, p<0.001, η^2^=0.33). The post hoc analysis further confirmed that the pain intensity ratings and self-unpleasantness ratings were higher under the general painful and sadistic painful conditions than under the neutral conditions in both treatment groups (ps<0.001). In addition, the difference between the general painful and sadistic painful conditions was larger in the OT group ((pain intensity: mean difference (MD)=0.37, p=0.039; self-unpleasantness: MD=0.74, p=0.009) than in the PL group (pain intensity: MD=-0.21, p=0.398; self-unpleasantness: MD=0.06, p=0.821). The differences in pain intensity ratings both between sadistic painful and neutral conditions and between general painful and neutral conditions were enhanced by the OT (sadistic painful vs. neutral: F(1,78)=11.73, p=0.001, η^2^=0.13; general painful vs. neutral: F(1,78)=4.80, p=0.03, η^2^=0.06). The differences in the self-unpleasantness ratings between sadistic painful and neutral conditions were also enhanced by the OT (F(1,78)=4.78, p=0.03, η^2^=0.06), but not the general painful vs. neutral conditions (F(1,78)=0.81, p=0.37, η^2^=0.01).

**References**

1 Pascual-Marqui, R. D. Standardized low-resolution brain electromagnetic tomography (sLORETA): technical details. *Methods. Find. Exp. Clin. Pharmacol.* **24**, 5-12 (2002).

2 Kosfeld, M., Heinrichs, M., Zak, P. J., Fischbacher, U. & Fehr, E. Oxytocin increases trust in humans. *Nature* **435**, 673-676; DOI:10.1038/nature03701 (2005).

3 De Dreu, C. K. W. *et al.* The neuropeptide oxytocin regulates parochial altruism in intergroup conflict among humans. *Science* **328**, 1408-1411; DOI:10.1126/science.1189047 (2010).

4 Ma, Y. N., Liu, Y., Rand, D. G., Heatherton, T. F. & Han, S. H. Opposing oxytocin effects on intergroup cooperative behavior in intuitive and reflective minds. *Neuropsychopharmacol* **40**, 2379-2387; DOI:10.1038/npp.2015.87 (2015).

5 Rosenberg, M. *Society and the adolescent self-image*. Vol. 11 (Princeton university press Princeton, NJ, 1965).

6 Adler, N. E. & Ostrove, J. M. Socioeconomic status and health: What we know and what we don't. *Ann. Ny. Acad. Sci.* **896**, 3-15; DOI:10.1111/j.1749-6632.1999.tb08101.x (1999).

7 Diener, E., Emmons, R. A., Larsen, R. J. & Griffin, S. The satisfaction with life scale. *J. Pers. Assess.* **49**, 71-75; DOI:10.1207/s15327752jpa4901_13 (1985).

8 Davis, M. H. Measuring individual differences in empathy: Evidence for a multidimensional approach. *J. Pers. Soc. Psychol.* **44**, 113-126 (1983). 9 Luo, S. Y., Yu, D. & Han, S. H. Genetic and neural correlates of romantic relationship satisfaction. *Soc. Cogn**. Affect. Neur.* **11**, 337-348; DOI:10.1093/scan/nsv117 (2016).

10 Preacher, K. J. & Hayes, A. F. Asymptotic and resampling strategies for assessing and comparing indirect effects in multiple mediator models. *Behav. Res. Methods.* **40**, 879-891; DOI:10.3758/Brm.40.3.879 (2008).

11 Mackinnon, D. P., Lockwood, C. M. & Williams, J. Confidence limits for the indirect effect: distribution of the product and resampling methods. *Multivariate. Behav. Res.* **39**, 99; DOI:10.1207/s15327906mbr3901_4 (2004).

12 Shrout, P. E. & Bolger, N. Mediation in experimental and nonexperimental studies: new procedures and recommendations. *Psychol. Methods.* **7**, 422-445 (2002).

13 Preacher, K. J., Rucker, D. D. & Hayes, A. F. Addressing moderated mediation hypotheses: theory, methods, and prescriptions. *Multivariate Behav Res* **42**, 185-227; DOI:10.1080/00273170701341316 (2007).

14 Efron B. The Jackknife, the Bootstrap and Other Resampling Plans. *Society for Industrial and Applied Mathematics* (1982).

15 Aiken, L. S. & West, S. G. Multiple regression: testing and interpreting interactions. *J. Oper. Res. Soc.* **45,** 119-120 (1994).

Table S1. Subjective information about the two genotype groups

| Variable | G/G | A/A | *F* | *p* | Partial η^2^ |
| --- | --- | --- | --- | --- | --- |
| Age (years) | 21.46±2.26 | 21.83±2.70 | 0.27 | 0.60 | 0.006 |
| Self-esteem | 29.42±4.37 | 30.63±3.95 | 1.01 | 0.32 | 0.03 |
| Life satisfaction | 22.21±6.87 | 23.63±6.57 | 0.53 | 0.47 | 0.01 |
| IRI | 69.67±10.70 | 71.04±10.78 | 0.20 | 0.66 | 0.004 |
| EC | 18.88±3.77 | 18.92±3.76 | 0.001 | 0.97 | 0.00 |
| PT | 17.46±3.19 | 18.75±3.65 | 1.70 | 0.20 | 0.04 |
| FS | 17.83±5.50 | 18.58±3.88 | 0.30 | 0.59 | 0.006 |
| PD | 15.50±3.30 | 14.79±3.43 | 0.53 | 0.47 | 0.01 |

Table S2. Subjective information about the two treatment groups

| Variable | OT Group | PL Group | *F* | *p* | Partial η^2^ |
| --- | --- | --- | --- | --- | --- |
| Age (years) | 19.75±1.13 | 19.83±1.65 | 0.06 | 0.81 | 0.001 |
| Self-esteem | 29.27±4.18 | 30.57±3.64 | 2.21 | 0.14 | 0.03 |
| Life satisfaction | 23.50±3.82 | 25.36±4.46 | 4.02 | 0.05 | 0.05 |
| SES | 5.11±2.46 | 5.48±1.32 | 1.36 | 0.25 | 0.02 |
| IRI | 66.43±9.23 | 65.50±8.09 | 0.23 | 0.64 | 0.003 |
| EC | 17.70±3.44 | 17.25±2.91 | 0.40 | 0.53 | 0.005 |
| PT | 17.72±3.50 | 17.40±3.02 | 0.20 | 0.66 | 0.003 |
| FS | 16.40±4.39 | 16.93±4.09 | 0.31 | 0.58 | 0.004 |
| PD | 14.60±3.24 | 13.92±2.68 | 1.03 | 0.31 | 0.01 |

Table S3. Results of the mediation analysis of the treatment effect on context-dependent empathic responses, with empathic responses to sadistic painful conditions as the mediator

| **Variable** | **Coeff** | **SE** | **t** | **p** |
| --- | --- | --- | --- | --- |
| **IV to Mediators** |  |  |  |  |
| Treatment | 0.86** | 0.29 | 2.99 | 0.004 |
| Dependent: Empathic responses for the sadistic painful condition |  |  |  |  |
|  |  |  |  |  |
| **Direct Effects of Mediator on DV** |  |  |  |  |
| Empathic responses for the sadistic painful condition | 0.41*** | 0.09 | 4.49 | <0.001 |
| Dependent: Context-dependent empathic responses |  |  |  |  |
|  |  |  |  |  |
| **Total Effect of IV on DV** |  |  |  |  |
| Treatment | 0.44 | 0.26 | 11.67 | 0.099 |
| Dependent: Context-dependent empathic responses |  |  |  |  |
|  |  |  |  |  |
| **Remaining Direct Effect of IV on DV** |  |  |  |  |
| Treatment | 0.08 | 0.25 | 0.33 | 0.75 |
| Dependent: Context-dependent empathic responses |  |  |  |  |
|  |  |  |  |  |
| Bias-Corrected Confidence Intervals |  |  | **LLCI95** | **ULCI95** |
| **Indirect effect of the treatment on context-dependent empathic responses via empathic responses for the sadistic painful condition** | | | | |
|  |  |  | 0.1155 | 0.8562 |

*p<0.05, **p<0.01, ***p<0.001

Notes: Confidence intervals for the indirect effect are bias-corrected and accelerated; bootstrap resamples=5000.

Table S4. Results of the mediation analysis of the effect of the treatment on context-dependent empathic responses, with empathic responses for the general painful condition as the mediator

| **Variable** | **Coeff** | **SE** | **t** | **p** |
| --- | --- | --- | --- | --- |
| **IV to Mediators** |  |  |  |  |
| Treatment | 0.42 | 0.29 | 1.47 | 0.15 |
| Dependent: Empathic responses for the general painful condition |  |  |  |  |
|  |  |  |  |  |
| **Direct Effects of Mediator on DV** |  |  |  |  |
| Empathic responses for the general painful condition | -0.41*** | 0.09 | -4.48 | <0.001 |
| Dependent: Context-dependent empathic responses |  |  |  |  |
|  |  |  |  |  |
| **Total Effect of IV on DV** |  |  |  |  |
| Treatment | 0.44 | 0.26 | 11.67 | 0.099 |
| Dependent: Context-dependent empathic responses |  |  |  |  |
|  |  |  |  |  |
| **Remaining Direct Effect of IV on DV** |  |  |  |  |
| Treatment | 0.61* | 0.24 | 2.57 | 0.01 |
| Dependent: Context-dependent empathic responses |  |  |  |  |
|  |  |  |  |  |
| Bias-corrected Confidence Intervals |  |  | **LCI95** | **UCI95** |
| **Indirect effect of treatment on context-dependent empathic responses via empathic responses for the sadistic painful condition** | | | | |
|  |  |  | -0.4721 | 0.0381 |

*p<0.05, **p<0.01, ***p<0.001

Notes: Confidence intervals for the indirect effect are bias-corrected and accelerated; bootstrap resamples=5000

Table S5. Results of a hierarchical regression analysis of the treatment and IRI scores on context-dependent empathic responses in Experiment 2

|  | Step1β | Step2β |
| --- | --- | --- |
| Treatment | 0.19 | 0.20 |
| IRI | -0.03 | -0.08 |
| Treatment×IRI |  | 0.38^***^ |
| R^2^ change | 0.04 | 0.14 |
| F change | 1.41 | 12.99^***^ |
| R^2^ | 0.04 | 0.18 |
| Adjusted R^2^ | 0.01 | 0.14 |
| Overall F | 1.41 | 5.42^**^ |
| df | 77 | 76 |

^**^ p<0.01, ^***^ p≤0.001

Table S6. Sadism-related ratings of stimuli

|  | Placebo Group | Oxytocin Group |
| --- | --- | --- |
| Neutral | 1.39±0.59 | 1.33±0.59 |
| General Pain | 2.26±1.25 | 2.01±1.13 |
| Sadistic Pain | 8.38±0.94 | 8.62±0.74 |


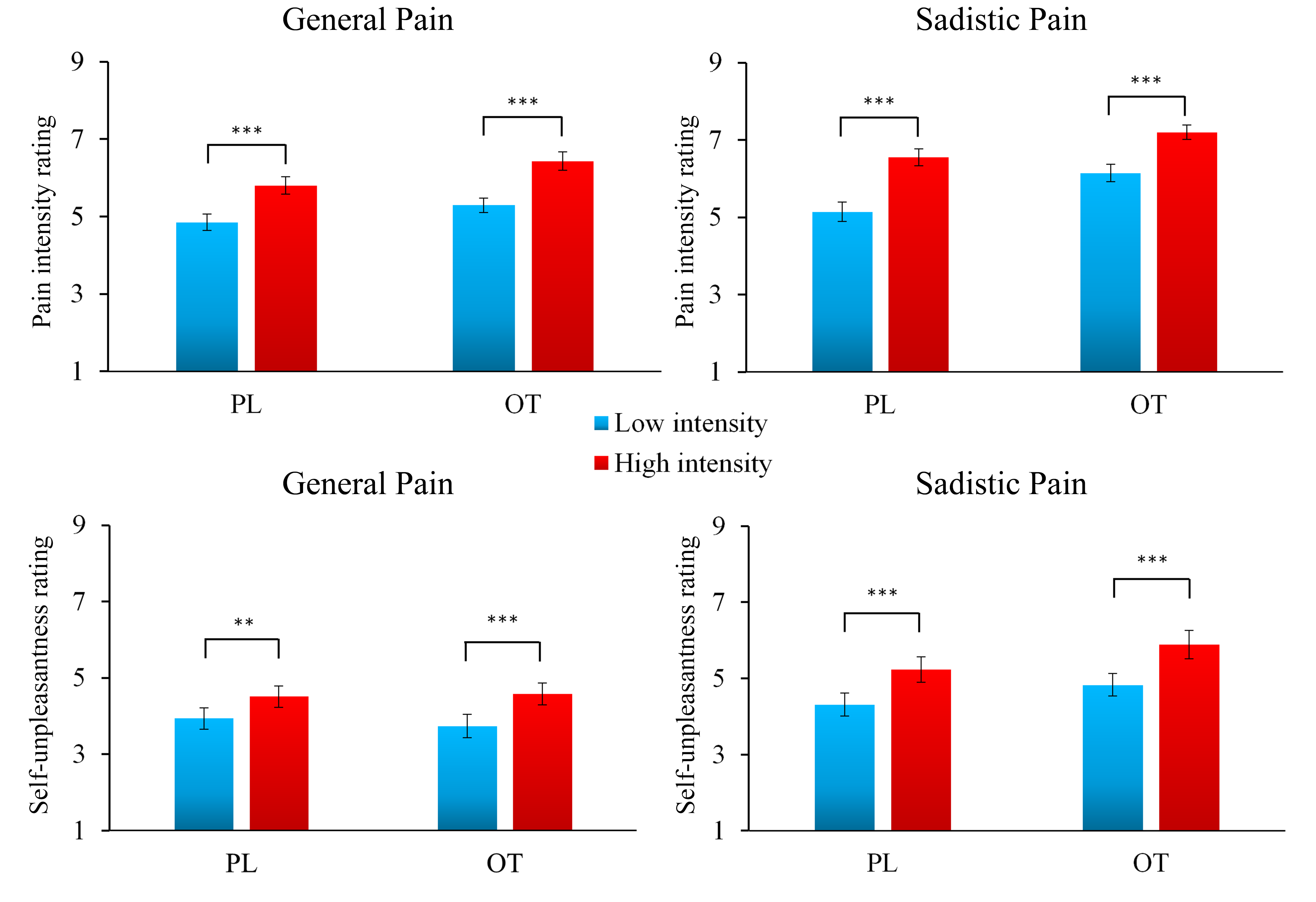


Figure S1. Mean pain intensity/self-unpleasantness rating for low pain intensity and high pain intensity stimuli reported by the two treatment groups. Error bars represent standard errors.


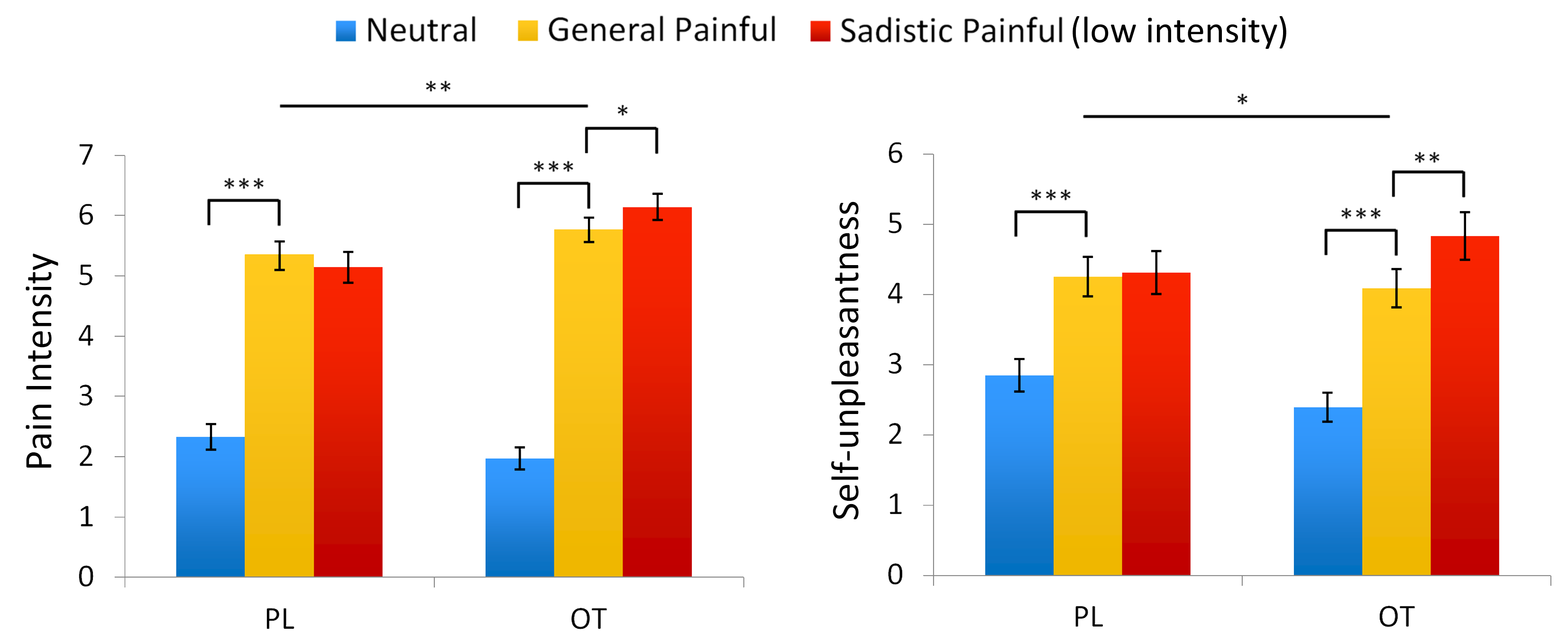


Figure S2. Mean pain intensity/self-unpleasantness rating scores in two treatment groups. Error bars represent standard errors.
